# Defining the structural basis for human leukocyte antigen reactivity in clinical transplantation

**DOI:** 10.1038/s41598-020-75355-4

**Published:** 2020-10-27

**Authors:** Yue Gu, Robynne W. K. Koh, May Ling Lai, Denise Pochinco, Rachel Z. C. Teo, Marieta Chan, Tanusya M. Murali, Chong Wai Liew, Yee Hwa Wong, Nicholas R. J. Gascoigne, Kathryn J. Wood, Julien Lescar, Peter Nickerson, Paul A. MacAry, Anantharaman Vathsala

**Affiliations:** 1 Department of Microbiology and Immunology, Yong Loo Lin School of Medicine, National University of Singapore, Singapore, Singapore; 2 CREATE Inflammatory Diseases Programme, Singapore, Singapore; 3 NUS Graduate School for Integrative Sciences and Engineering, National University of Singapore, Singapore, Singapore; 4 National University Centre for Organ Transplantation (NUCOT), National University Hospital, Singapore, Singapore; 5 Center for Transfusion Medicine, Health Sciences Authority, Singapore, Singapore; 6 Transplant Immunology Laboratory, Shared Health Inc., Winnipeg, MB Canada; 7 NTU Institute of Structural Biology, Nanyang Technological University, Singapore, Singapore; 8 School of Biological Sciences, Nanyang Technological University, Singapore, Singapore; 9 Transplantation Research Immunology Group, Nuffield Department of Surgical Sciences, University of Oxford, Oxford, UK; 10 Department of Medicine, University of Manitoba Max Rady College of Medicine, Winnipeg, MB Canada; 11 Division of Nephrology, Department of Medicine, Yong Loo Lin School of Medicine, National University of Singapore, Singapore, Singapore

**Keywords:** Immunology, Microbiology, Medical research, Nephrology

## Abstract

The current state-of-the-art technology employed to assess anti-human leukocyte antigen antibodies (Anti-HLA Ab) for donor-recipient matching and patient risk stratification in renal transplantation is the single antigen bead (SAB) assay. However, there are limitations to the SAB assay as it is not quantitative and due to variations in techniques and reagents, there is no standardization across laboratories. In this study, a structurally-defined human monoclonal alloantibody was employed to provide a mechanistic explanation for how fundamental alloantibody biology influences the readout from the SAB assay. Performance of the clinical SAB assay was evaluated by altering Anti-HLA Ab concentration, subclass, and detection reagents. Tests were conducted in parallel by two internationally accredited laboratories using standardized protocols and reagents. We show that alloantibody concentration, subclass, laboratory-specific detection devices, subclass-specific detection reagents all contribute to a significant degree of variation in the readout. We report a significant prozone effect affecting HLA alleles that are bound strongly by the test alloantibody as opposed to those bound weakly and this phenomenon is independent of complement. These data highlight the importance for establishing international standards for SAB assay calibration and have significant implications for our understanding of discordance in previous studies that have analyzed its clinical relevance.

## Introduction

Donor-specific antibodies (DSA) targeting mismatched HLA molecules on donor organs is associated with antibody-mediated rejection (AMR), graft dysfunction, and impaired long-term survival among organ transplant recipients^1–8^. As effective therapies for AMR are lacking^9,10^, the detection of early DSA responses and diagnosis of AMR are currently the main approaches while novel strategies such as epitope matching aim to prevent AMR by reducing recipient to donor disparity.

The state-of-the-art technology employed to assess Anti-HLA Ab in the clinical context is the SAB assay. In this assay, patient serum is incubated with a pool of single HLA antigen-coated beads and the binding of alloantibodies to individual HLA alleles translated into a normalized mean fluorescence intensity (MFI) value^11^. The MFI readout reflects: (1) the broad reactivity of Anti-HLA Ab across HLA alleles due to a high degree of homology; (2) a semi-quantitative measure of the amount of antibody bound to the beads that can be used to establish a threshold for clinical action. The assay is widely utilized as a prognostic test for patient risk stratification, HLA-compatible donor identification, employed to monitor DSA development in transplant recipients, and is crucial for the diagnosis of AMR^12^. Thus, the SAB assay plays a critical role in clinical decision making pre- and post-transplantation^13^.

However, there are limitations in assay performance due to the conformational status of the HLA molecules on the beads, variations in anti-IgG detection reagents used, alternative techniques employed by different laboratories, and other vendor-specific kit issues^14^. Thus, many studies have highlighted significant variations in the normalized MFIs for matching allosera samples tested in internationally accredited clinical laboratories employing standardized reagents and protocols^13,15^.

Another limitation of the assay is the ‘prozone’ or ‘hook’ effect, which results in false negatives whereby patients with high titers of Anti-HLA Ab have a low or undetectable MFI signature^16–18^. This phenomenon has often been attributed to complement inhibition of DSA binding to the HLA-coated beads^19,20^ or to IgM or other blocking antibodies^16,21^. Despite these limitations, the SAB assay is the principal method for assessing Anti-HLA Ab in patients’ serum^13^.

Another approach to minimize DSA generation and hence chronic AMR and associated graft failure, is epitope matching^22–24^. A key component of epitope matching is the ‘eplet’ which is defined by a small number of polymorphic residues, usually within a radius of 3–3.5 Å in an antibody-accessible location on the HLA molecule^25^. An extensive database including both antibody-verified eplets and those predicted by in silico modeling^26^ is a useful tool for ascertaining the eplets on HLA targeted by Anti-HLA Ab. However, analysis of eplets as derived from alloantibodies found in polyclonal sera is nevertheless confounded by variations intrinsic to the SAB assay highlighted earlier. Thus, the potential applicability of such in silico predictive algorithms remains limited by the scarcity of appropriate training data.

These problems may be overcome by the use of human monoclonal Anti-HLA Abs to better define paratope-epitope interactions. We have previously characterized an alloantibody termed 2E3 that targets the antibody-verified eplet 90D and solved the paratope-epitope structure of 2E3-HLA-A*11:01 complex by X-ray crystallography. 2E3 was engineered into the four major human IgG subclasses^27^. In this study, we analyze the HLA reactivity patterns of the different IgG subclasses of 2E3 at varying concentrations using the clinical SAB assay. We hereby provide a scientific basis for the MFI readouts derived from this assay and evaluate the influence of commonly proposed variables such as alloantibody concentration and subclass. Finally, we have conducted a thorough analysis of the prozone effect and observe that this is more likely to impact upon HLA alleles that are bound with high affinity by the alloantibody as opposed to those where the binding events are of intermediate or weak affinity. These data represent the first detailed functional appraisal of the SAB assay using a structurally defined monoclonal human alloantibody.

## Materials and methods

### Antibody testing using the SAB assay

Human monoclonal antibody 2E3 was isolated by panning the HumanyxI phage-Fab library against recombinant refolded HLA-A*11:01, engineered, purified, and characterized as previously described^27^.

Purified monoclonal antibodies were transported to the clinical laboratories on dry ice. Tests on four IgG subclasses of 2E3 were performed using LABScreen Single Antigen Class I beads (LS1A04 from One Lambda Inc., Canoga Park, CA) according to the manufacturer’s protocol. For each test, 1 µL of 2E3 was diluted with 19 µL of Negative Control Serum (LS-NC from One Lambda Inc., Canoga Park, CA) to achieve the final concentration.

2E3-IgG1 was tested on the SAB assay with and without ethylenediaminetetraacetic acid (EDTA) treatment. 1 μL of 0.1625 M EDTA solution was added to 26.25 μL of each respective sample mixture. 20 µL of the resulting sample mixture was incubated with the beads for 30 min at room temperature in the dark. The samples were then washed, and phycoerythrin (PE)-labeled polyclonal goat anti-human IgG antibody (LS-AB2 from One Lambda Inc., Canoga Park, CA) diluted at 1:100 was added. After a second incubation step, samples were washed three times, and signal was detected on Luminex 200 at Health Science Authority (HSA), Singapore, or by LABScan 3D (One Lambda) at Shared Health Inc. (SHI), Canada. A normalized trimmed MFI value was reported for each bead each test.

### Subclass-specific antibody testing

Purified 2E3 antibodies were diluted in Negative Control Serum and incubated with LABScreen Single Antigen Class I beads as mentioned above. After washing, 100 µL of PE-labeled monoclonal murine anti-human detection antibodies specific for IgG1, IgG2, IgG3 or IgG4 were added to the sample mixture. All secondary antibodies were a generous gift from One Lambda. Signal detection and normalization of MFI values were performed as above.

### Statistical analysis

Raw MFI values were normalized using the following formula: (Sample #N beads—sample negative control beads)—(Negative control #N beads—Negative control beads). One-way ANOVA Kruskal–Wallis test was used for statistical analysis between reactive alleles (N = 15), cross-reactive alleles (N = 18), non-reactive allele (N = 60), followed by Dunn’s multiple comparisons test. Data are plotted as mean with 95% confidence interval (CI).

## Results

### SAB assay detection of a structurally-defined human alloantibody

A human alloantibody 2E3 was engineered into four IgG subclasses, and allele reactivity corresponded to eplet 90D was confirmed by X-ray crystallography. We spiked 2E3-IgG1 into the negative control human serum utilized for the clinical SAB assay—the final concentrations tested were 0.02 μg/mL, 1 μg/mL, 2 μg/mL, 10 μg/mL and 50 μg/mL, respectively. The HLA-binding pattern was assayed by two internationally accredited clinical laboratories: HSA, Singapore and SHI, Canada. All tests were conducted in parallel.

**Figure 1:**
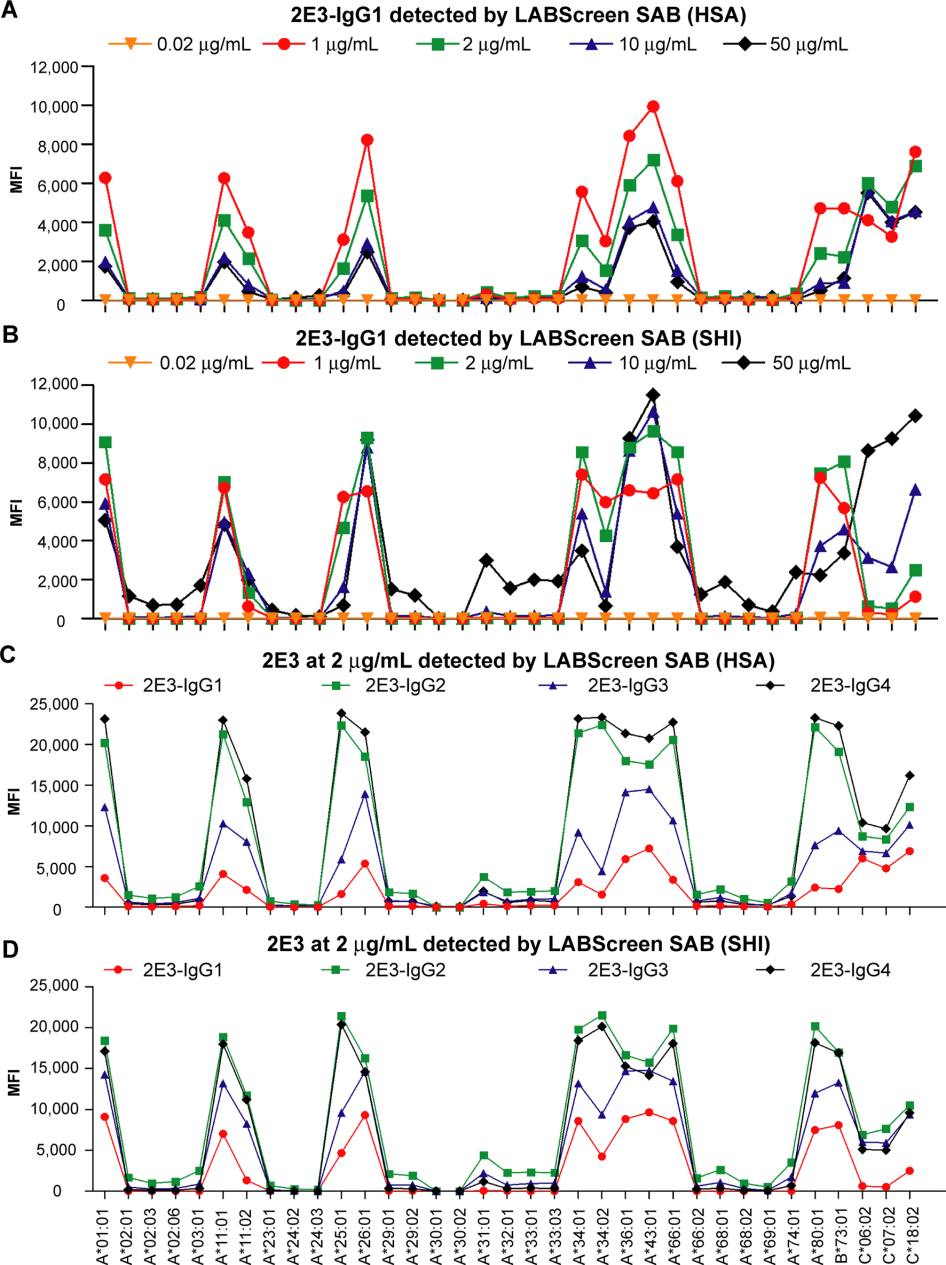
Antibody 2E3 detected by the SAB assay. 2E3-IgG1 was diluted in negative control serum to achieve final concentrations of 0.02 μg/mL, 1 μg/mL, 2 μg/mL, 10 μg/mL and 50 μg/mL, and tested by accredited laboratories (**A**) HSA and (**B**) SHI. All HLA-A alleles, 2E3-reactive HLA-B and HLA-C alleles are shown in the figure. Four human IgG subclasses of 2E3 were tested at 2 μg/mL by (**C**) HSA and (**D**) SHI. *SAB* single antigen beads; *MFI* mean fluorescence intensity; *HSA* Heath Sciences Authority, Singapore; *SHI* Shared Health Inc., Canada; *HLA* human leukocyte antigen.

Both laboratories reported negligible MFI values (< 50) when 2E3-IgG1 was tested at 0.02 μg/mL. At 2E3-IgG1 concentrations of 1 μg/mL, 2 μg/mL and 10 μg/mL, the list of reactive alleles correlated well with eplet 90D (Fig. 1A,B). When 2E3-IgG1 was employed at 50 μg/mL and tested by SHI, we observed cross-reactivity to most HLA-A antigens but not HLA-B or HLA-C antigens (Fig. 1B). Depending on the allele analyzed, MFI readouts from HSA were generally lower than those from SHI. Based on our exclusion of other variables, this is linked to differences in the instrumentation employed to detect fluorescent signatures and/or small variations in technique employed by operators at both sites.

Based on the allele reactivity of 2E3 and previous structural studies, we categorized the 97 HLA Class I antigens utilized into three groups (Table 1). Group 1, 2E3 reactive alleles, refers to HLA Class I antigens to which 2E3 binds at low concentrations and includes those with eplet 90D (except HLA-C*04:01, discussed previously^27^). Group 2, 2E3 cross-reactive alleles, refers to Class I antigens that 2E3 binds weakly at high antibody concentrations and includes most HLA-As that are not in Group 1 (except HLA-A*30:01 and HLA-A*30:02, discussed previously^27^). Group 3, 2E3 non-reactive alleles, refers to all remaining HLA Class I antigens to which 2E3 does not bind. The MFI readouts of these alleles were below 100 at a high 2E3 concentration of 50 μg/mL (Fig. 1B).

**Table 1:** List of HLA Class I Luminex alleles based on binding activity of antibody 2E3.

	Definition	HLA Class I Luminex alleles
Reactive alleles	2E3 binds to these alleles at relatively low antibody concentrations No or insufficient structural evidence to suggest significantly disrupted interactions between 2E3 and these alleles	A*01:01, A*11:01, A*11:02, A*25:01, A*26:01, A*34:01, A*34:02, A*36:01, A*43:01, A*66:01, A*80:01, B*73:01, C*06:02, C*07:02, C*18:02
Cross-reactive alleles	2E3 only binds to these alleles at high antibody concentrations Supported by structural analysis	A*02:01, A*02:03, A*02:06, A*03:01, A*23:01, A*24:02, A*24:03, A*29:01, A*29:02, A*31:01, A*32:01, A*33:01, A*33:03, A*66:02, A*68:01, A*68:02, A*69:01, A*74:01
Non-reactive alleles	2E3 does not bind to these alleles even at high antibody concentrations Supported by structural analysis	A*30:01, A*30:02, B*07:02, B*08:01, B*13:01, B*13:02, B*14:01, B*14:02, B*15:01, B*15:02, B*15:03, B*15:10, B*15:11, B*15:12, B*15:13, B*15:16, B*18:01, B*27:05, B*27:08, B*35:01, B*37:01, B*38:01, B*39:01, B*40:01, B*40:02, B*40:06, B*41:01, B*42:01, B*44:02, B*44:03, B*45:01, B*46:01, B*47:01, B*48:01, B*49:01, B*50:01, B*51:01, B*51:02, B*52:01, B*53:01, B*54:01, B*55:01, B*56:01, B*57:01, B*57:03, B*58:01, B*59:01, B*67:01, B*78:01, B*81:01, B*82:01, C*01:02, C*02:02, C*03:02, C*03:03, C*03:04, C*04:01, C*05:01, C*08:01, C*12:03, C*14:02, C*15:02, C*16:01, C*17:01

### Influence of alloantibody IgG subclass on the SAB assay

**Figure 2:**
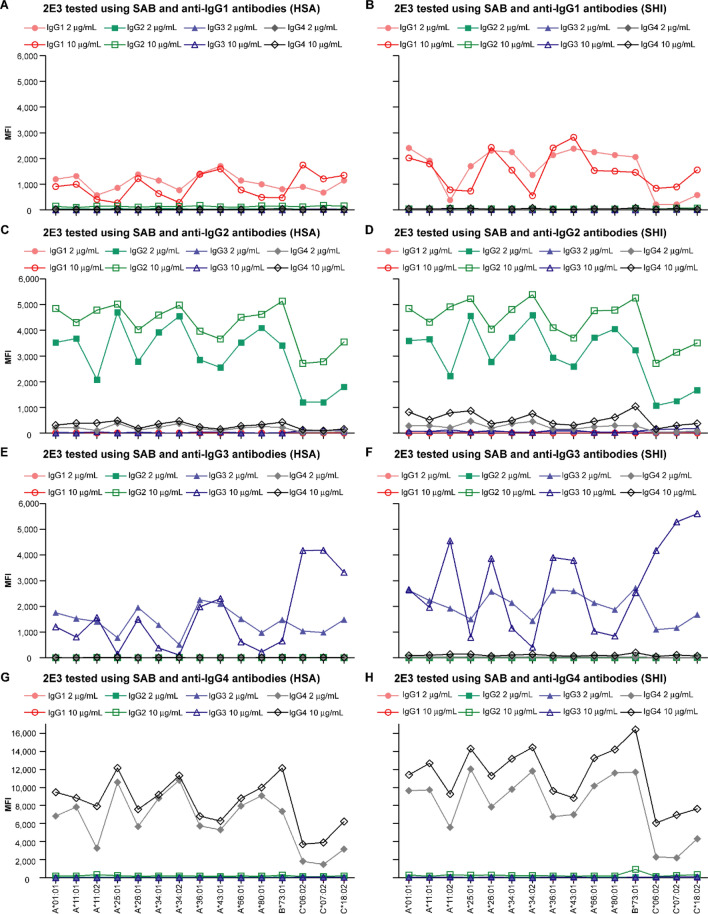
Anti-IgG2 detection antibodies demonstrate cross-reactivity with 2E3-IgG4. Antibody 2E3 was recombinantly expressed as the four human IgG subclasses and tested at 2 μg/mL and 10 μg/mL using the SAB assay. The assays were performed by substituting the standard pan-IgG detection antibodies with (**A**, **B**) IgG1-specific, (**C**, **D**) IgG2-specific, (**E**, **F**) IgG3-specific, or (**G**, **H**) IgG4-specific detection reagents. 2E3-reactive HLA Class I alleles are shown. Figure illustrates assay performance at both laboratories. *SAB* single antigen beads, *MFI* mean fluorescence intensity, *HLA* human leukocyte antigen.

Four IgG subclasses of 2E3 were tested at final concentrations of 2 μg/mL and 10 μg/mL. The results show that with phycoerythrin (PE)-labelled anti-human IgG (Pan-IgG) detection, MFI values ranged as follows: 2E3-IgG2 ≈ 2E3-IgG4 > 2E3-IgG3 > 2E3-IgG1 (Fig. 1C,D). The pattern of reactivity, as reflected by MFIs, were similar for 2E3-IgG1 and 2E3-IgG3 and for 2E3-IgG2 and 2E3-IgG4 respectively (Fig. 1C,D, Fig. S1). When the four 2E3 IgG subclasses were assayed using *subclass-specific* detection reagents, all four detection antibodies were able to recognize their specific 2E3 subclass (Fig. 2). However, 2E3-IgG4 was detected by the anti-IgG2 detection antibody, though at a lower MFI compared to 2E3-IgG2, implying a degree of cross-reactivity (Fig. 2C,D). All four IgG subclasses were detected at a lower MFI using the subclass-specific detection antibodies compared to the Pan-IgG detection antibody (Fig. S2).

### Correlation between alloantibody binding strength and prozone effect

**Figure 3:**
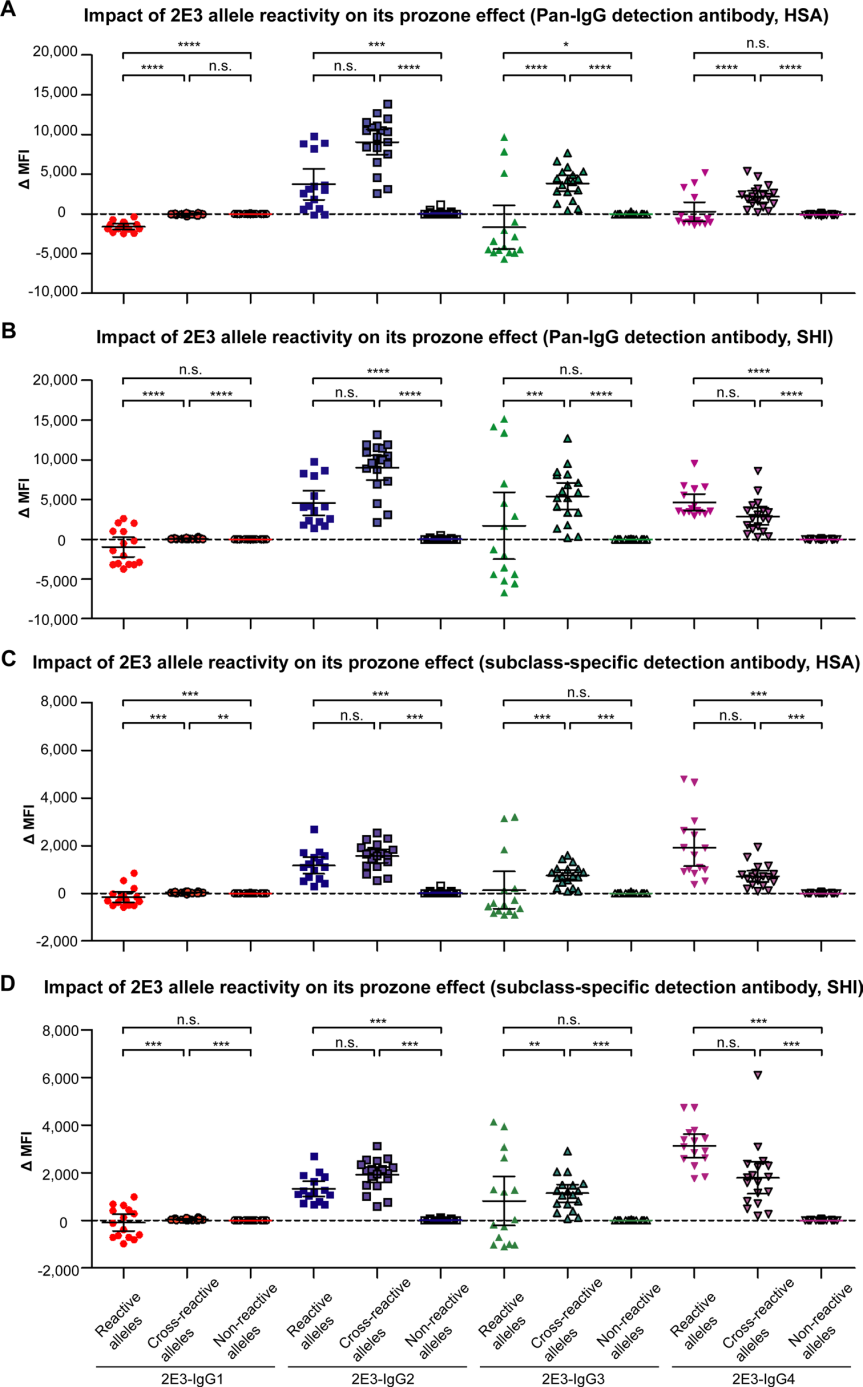
Impact of 2E3 binding reactivity on prozone effect observed in the SAB assay. The MFI difference (∆MFI) between 2E3 tested at 2 μg/mL and 2E3 tested at 10 μg/mL was calculated for each allele and plotted according to the 2E3 reactivity categories as classified in Table 1 and different 2E3 subclasses using (**A**, **B**) Pan-IgG detection reagent, and (**C**, **D**) corresponding subclass-specific detection reagents**.** Data represented as mean with 95% CI. Statistical significance between reactive (N = 15), cross-reactive (N = 18) and non-reactive categories of the same subclass (N = 64) tested at the same center was calculated by one-way ANOVA Kruskal–Wallis tests followed by Dunn’s multiple comparisons test. Figure illustrates results from both laboratories. *SAB* single antigen beads, *MFI* mean fluorescence intensity, *HLA* human leukocyte antigen, *CI* confidence interval, *ANOVA* analysis of variance.

In this study, we employed ∆MFI which depicts the MFI change from 2E3 at 2 μg/mL and 10 μg/mL, as an indicator of the prozone effect. ∆MFI was individually calculated for each HLA allele for each 2E3 subclass. We plotted the ∆MFI value according to 2E3 allele reactivity groups and 2E3 subclasses (Fig. 3A,B). When 2E3-IgG1 or 2E3-IgG3 were tested, over 50% of the reactive alleles had negative ∆MFI values. Correspondingly, ∆MFI of the 2E3-IgG1 reactive alleles was significantly lower than that of the 2E3-IgG1 cross-reactive alleles (p < 0.001), suggesting a distinct prozone effect. Similar trends were observed for 2E3-IgG3 and 2E3-IgG4 tested at HSA, as well as 2E3-IgG3 tested at SHI (p < 0.001). Although the mean ∆MFI of 2E3-IgG2 reactive alleles were lower than that of cross-reactive alleles, the differences were not statistically significant due to the wide range of ∆MFI values. Similar findings were observed when 2E3 binding was measured by the corresponding subclass-specific detection reagents (Fig. 3C,D). This analysis clearly demonstrates the prozone effect specifically for reactive alleles but not for cross-reactive alleles where 2E3 binds weakly.

**Figure 4:**
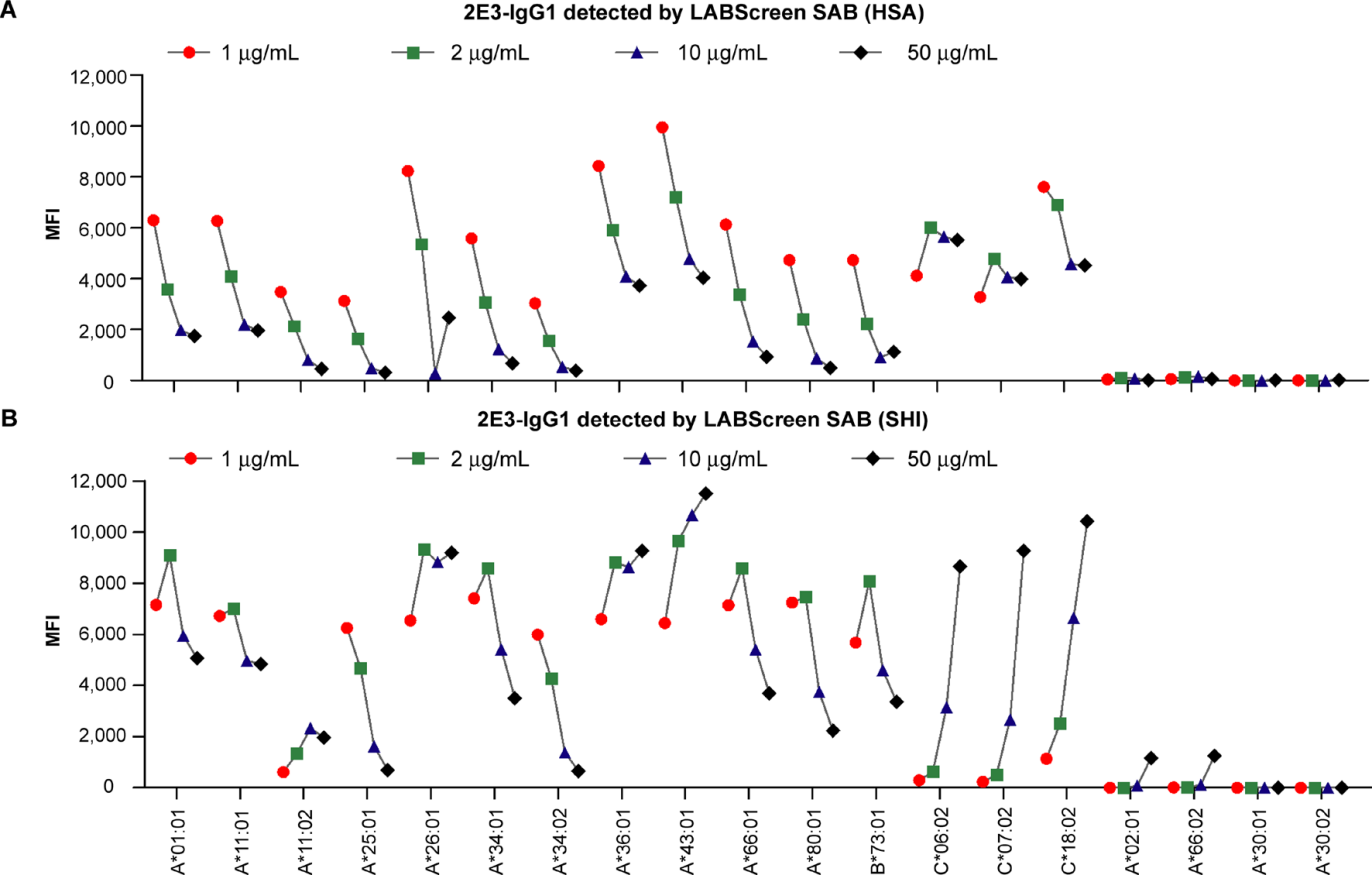
Prozone effect observed for 2E3-IgG1 at increasing concentrations as identified by the SAB assay. MFI of 2E3-IgG1 at 1 μg/mL, 2 μg/mL, 10 μg/mL and 50 μg/mL were connected with lines and plotted against the allele for both tests performed (**A**) at HSA and (**B**) at SHI. All 2E3-reactive HLA Class I alleles, two cross-reactive alleles (A*02:01 and A*66:02) and two non-reactive alleles (A*30:01 and A*30:02) are shown in the figure. *SAB* single antigen beads; *MFI* mean fluorescence intensity; *HSA* Heath Sciences Authority, Singapore; *SHI* Shared Health Inc., Canada; *HLA* human leukocyte antigen.

We plotted the MFI values at increasing concentrations from 1 to 50 μg/mL for all 2E3 reactive alleles, two 2E3 cross-reactive HLA-A alleles, and two 2E3 non-reactive HLA-A alleles (Fig. 4). MFI values measured at HSA showed a decreasing trend from 1 to 50 μg/mL for most 2E3 reactive alleles (Fig. 4A). Pre-treatment with EDTA was effective at restoring the MFI at 50 μg/mL but the decreasing trend of MFI from 1 to 10 μg/mL remained altered (Fig. S3). Results from SHI indicated that 2E3 reactive alleles had the highest MFI at 2 μg/mL, with MFIs decreasing at higher concentrations (Fig. 4B). As anticipated, the MFI values only become positive at 50 μg/mL for 2E3 cross-reactive alleles. Taken together, these results demonstrate the prozone effect consistently for reactive alleles to which 2E3 binds strongly.

## Discussion

We have previously reported a structural definition of an anti-HLA-A*11:01 human monoclonal alloantibody 2E3^27^. Using this alloantibody, we investigated the influence of antibody concentration, subclass, and allele reactivity on the readout from the principal clinical SAB assay used in transplantation. We report that the reactivity pattern of 2E3-IgG1 at concentrations from 1 to 10 μg/mL corresponds well with eplet 90D on HLA Class I alleles.

The lack of an international threshold for MFI values that define a DSA response is a key challenge in the analysis of SAB assay results. In addition to a lack of standardization of reagents across laboratories, lot to lot differences in antigen density, orientation and quality of antigen (native versus denatured) has also been reported^28–30^. These confound attempts to make the assay truly quantifiable. To evaluate the influence of Anti-HLA Ab concentration, 2E3-IgG1 was tested at five physiological concentrations from 0.02 to 50 μg/mL by two ASHI/CAP certified laboratories. Here, we show that the maximal MFI differs by 2000–4000 between the two laboratories despite our measures to control the principal variables (Fig. 1A,B). Our results suggest that differences in MFI can be explained by the employment of different analytic readers or by small variations in technique that are operator specific. These results highlight the current challenges in standardizing thresholds to define significance in the clinical context. Our approach offers the possibility of using a series of well characterized human monoclonal alloantibodies as calibrators to standardize readouts across laboratories and correlate these with alloantibody concentration.

Next, we sought to evaluate the influence of human IgG subclasses on the consistency in the SAB assay data from both laboratories. We chose concentrations of 2 μg/mL and 10 μg/mL since they yielded MFI values commonly observed in clinical settings and HLA-binding patterns that are consistent with other binding assays^27^. We show that different subclasses of the same alloantibody tested resulted in differential MFI values suggesting a degree of preferential binding of the Pan-IgG detection reagent employed for certain subtypes or changes in the binding characteristics of 2E3 that are subclass-specific (Fig. 1C,D).

Importantly, the subclass-specific detection reagents employed in this study performed well in distinguishing different IgG subclasses with the exception of anti-IgG2 (Fig. 2). The importance of different subclasses of Anti-HLA Ab has been highlighted in previous studies. Lefaucheur et al*.* described 55 and 33 transplant recipients with IgG2 DSA and IgG4 DSA respectively; of these, 28 cases (31.8%) had both IgG subclasses^31^. The actual proportion with IgG2 DSA could have been lower since those with IgG4 DSAs may have been inaccurately detected by the anti-IgG2 detection reagent. In a prospective study in which 851 patients were characterized for their DSAs in relation to allograft outcomes, Viglietti et al*.* demonstrated that IgG3 subclass positivity or complement binding capacity predicted kidney allograft loss better than the general (non-subclass-specific) detection of DSAs. Given the potential implications of IgG subclass identification for predicting outcomes in clinical transplantation, assays to better identify these are vitally important^32^. The fact that the detection antibody identified 2E3-IgG3 without evidence of cross-reactivity is reassuring and offers promise for clinical translation. Nevertheless, there is a risk of misidentifying IgG2 and IgG4 alloantibodies, a limitation that needs to be addressed separately. From our study, these subclass-specific secondary reagents give a significantly lower average MFI value in comparison to the Pan-IgG (Fig. S2). This difference may be due to a loss of linear signal amplification associated with employing monoclonal versus polyclonal antibody detection reagents^33^. As standardization across laboratories would be critical for detection of Anti-HLA Ab subclasses, the requirement for a series of subclass-specific calibrators as part of assay standardization is again underscored.

In this study, we observed two different trends of MFI values, one shared by 2E3-IgG1 and 2E3-IgG3, versus the other shared by 2E3-IgG2 and 2E3-IgG4 (Fig. 1C,D). It has been suggested that antibody constant region can alter the fine specificity and affinity of the antibody to its epitope^34^. We therefore propose that some changes in patient allosera allele reactivity over time, could be due to class switch of their existing alloantibodies, in addition to those attributed to affinity maturation or de novo alloantibody development.

The prozone effect has been yet another challenge in the interpretation of SAB assay results. Many clinical centers have reported this phenomenon for both HLA Class I and Class II SAB assays when testing polyclonal allosera^17,35^. It has been suggested that high concentrations of complement component 1 (C1) can competitively displace the detection antibodies, causing a reduced signal^21^. Pre-treatment of serum with dithiothreitol (DTT) or EDTA has been found to partially abrogate the prozone effect^19,20,36–38^.

We analyzed the prozone effect in two ways: (1) a comparison of ∆MFI values of reactive versus cross-reactive versus non-reactive alleles (Fig. 3); (2) An examination of the trend of MFI changes over four increasing concentrations of 2E3-IgG1 (Fig. 4). Collectively, these data show that the prozone effect is more likely to occur where alloantibody 2E3 binds strongly to specific alleles—as revealed by both structural analysis and MFI values. No prozone effect was observed for alleles bound weakly by 2E3.We also show that the prozone effect can occur where sample interference by complement is not a consideration (Fig. S3). At high concentrations, alloantibodies compete for binding to the limited number of antigens on the bead, a concept of bead saturation proposed by Tambur et al*.*^19^. It is also possible that partially folded or unfolded HLA present on the same bead as folded HLA^29^ may favor weak binding events by alloantibodies that are subsequently washed-off during the assay procedure. Moreover, at high concentrations, alloantibodies may bind to cross-reactive alleles, causing a dilution effect that reduces binding to antigen-coated beads for which they are more specific. These factors can all potentially create a prozone effect. A better understanding of structural relationships between HLA and Anti-HLA Ab will yield improved approaches to address this issue.

The prozone effect was particularly prominent among the majority of 2E3 reactive alleles when testing with IgG1 and IgG3 (Fig. 3), the two IgG subclasses capable of initiating downstream pathways such as complement-dependent cytotoxicity. In the clinical setting, this would translate to low MFI values being recorded at high antibody concentrations, with MFI values increasing upon titration of patient sera. Our data reinforces the current recommendation for testing titrated patient sera in the clinical context. Since the prozone effect was not observed for cross-reactive alleles, any drastic changes in MFI values or altered allele reactivity pattern on the SAB assay occurring with titration (i.e. high MFI alleles display low MFI and low MFI alleles display high MFI with titration) may serve as a warning of potential prozone effect.

In summary, whilst alloantibody-mediated graft injury is the leading cause of graft failure in organ transplantation, the current gold standard for alloantibody assessment with the SAB assay has limitations as a predictive biomarker for subsequent graft injury. While detailed analyses of the specificities of alloantibody obtained from sensitized patients, together with in silico modeling of antibody-epitope/eplet interactions has significantly advanced the field, progress in the clinical management of Anti-HLA Ab-mediated graft injury has been slow. The current state of the art does not allow us to predict with precision which HLA or epitope/eplet mismatches engender an alloimmune response or identify those DSAs that are potentially pathogenic or distinguish patients with DSA who will benefit from therapy.

The strategy described here details significant advances in this area on several fronts. Our structural approach can translate into a better elucidation of HLA antigens and their potential to incite an alloantibody response. Our demonstration of 2E3 binding to a 90D defined eplet on A*11:01 is corroborative of that predicted by Duquesnoy^39^, suggesting that this form of analysis could complement in silico modeling approaches such as HLAMatchmaker and Electrostatic Mismatch Score system as suggested by Mallon^40^. The use of defined human IgG subclasses offers opportunities for calibration of the standard and subclass-specific SAB assay across laboratories. Thus, a detailed structural definition of the paratope-epitope relationship between a human alloantibody and its target HLA can translate into a precise and systematic approach to defining immune reactivity in organ transplantation and guide organ allocation practices. Such an approach is envisioned to improve the prediction of pathogenicity of Anti-HLA Ab and permit a guided approach to their treatment so as to reduce allograft failure.

## Supplementary information

Supplementary Figures.
