# Supplementary material for: Defining the structural basis for human leukocyte antigen reactivity in clinical transplantation
Source: Sci Rep. 2020 Oct 27;10:18397. doi: 10.1038/s41598-020-75355-4 (PMC7591533; doi:10.1038/s41598-020-75355-4)
Supplement: Supplementary file 1 — Supplementary Figures. [file 41598_2020_75355_MOESM1_ESM.docx]

**Supplementary Materials** for manuscript titled

**Defining the structural basis for human leukocyte antigen reactivity in clinical transplantation**

**Authors:** Yue Gu^1,2,3^, Robynne W.K. Koh^4^, May Ling Lai^5^, Denise Pochinco^6^, Rachel Z.C. Teo^4^, Marieta Chan^5^, Tanusya M. Murali^1^, Chong Wai Liew^7^, Yee Hwa Wong^7,8^, Nicholas R.J. Gascoigne^1^, Kathryn J. Wood^9^, Julien Lescar^7,8^, Peter Nickerson^6,10^, Paul A. MacAry^1,2,3*†^, Anantharaman Vathsala^4,11*†^

Table of Contents

**Supplementary Figure 1.** Structural analysis of 2E3 allele specificity. **1**

**Supplementary Figure 2.** Subclass-specific detection reagents gave lower signal intensities than Pan-IgG detection antibodies. **2**

**Supplementary Figure 3.** Pre-treatment with EDTA did not fully abrogate the prozone effect. **4**


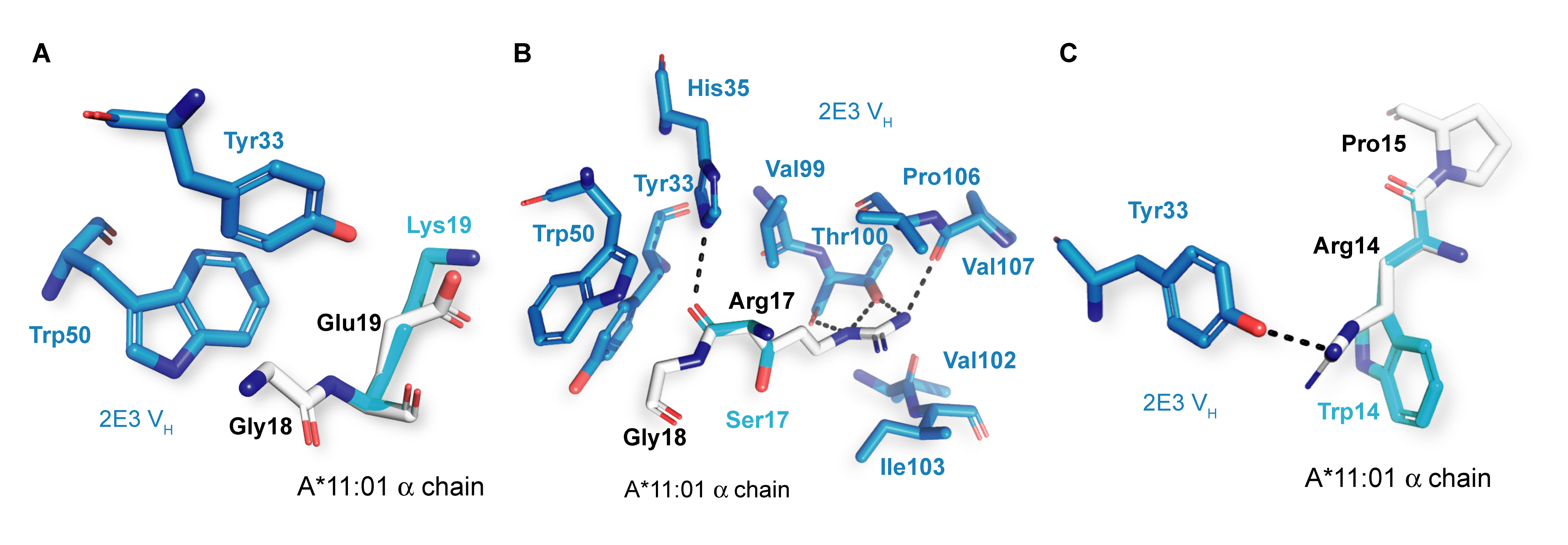


**Fig. S1.** Structural analysis of 2E3 allele specificity. **(A)** Magnified view of the interaction between HLA α chain position 19 and 2E3 V_H_ (blue). Glu19 in HLA-A*11:01 (white) was duplicated and mutated to Lys19 (cyan), which is in HLA-A*11:02. **(B)** Magnified view of the interaction between HLA α chain residue 17 and 2E3 V_H_ (blue). Arg17 in HLA-A*11:01 (white) was duplicated and mutated to Ser17 (cyan), which is in HLA-A*30:01 and HLA-A*30:02. **(C)** Magnified view of the interaction between HLA α chain residue 14 and 2E3 V_H_ (blue). Arg14 in HLA-A*11:01 (white) was duplicated and mutated to Trp14 (cyan), which is in HLA-C*04:01. V_H_: antibody heavy chain variable region.


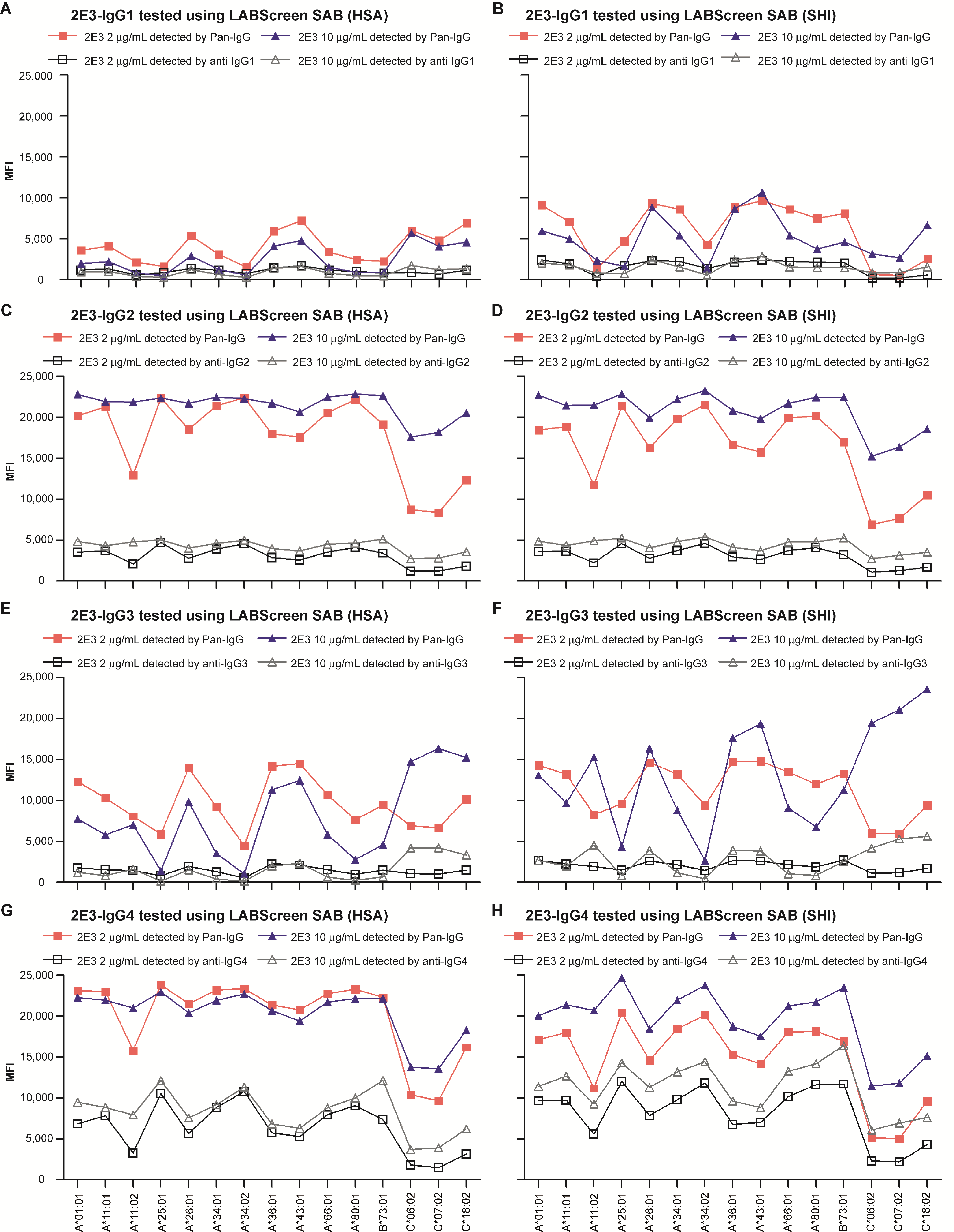


**Fig. S2.** Subclass-specific detection reagents gave lower signal intensities than Pan-IgG detection antibodies. Recombinantly expressed **(A, B)** 2E3-IgG1, **(C, D)** 2E3-IgG2, **(E, F)** 2E3-IgG3, and **(G, H)** 2E3-IgG4 were tested at 2 μg/mL and 10 μg/mL using the SAB assay. The assays were repeated by substituting the standard Pan-IgG detection antibodies with IgG subclass-specific detection reagents as secondary antibodies. 2E3-reactive HLA Class I alleles are shown. Figure illustrates assay performance at both laboratories. MFI: mean fluorescence intensity. HSA: Heath Sciences Authority, Singapore. SHI: Shared Health Inc., Canada. SAB: single antigen beads. HLA: human leukocyte antigen.

**
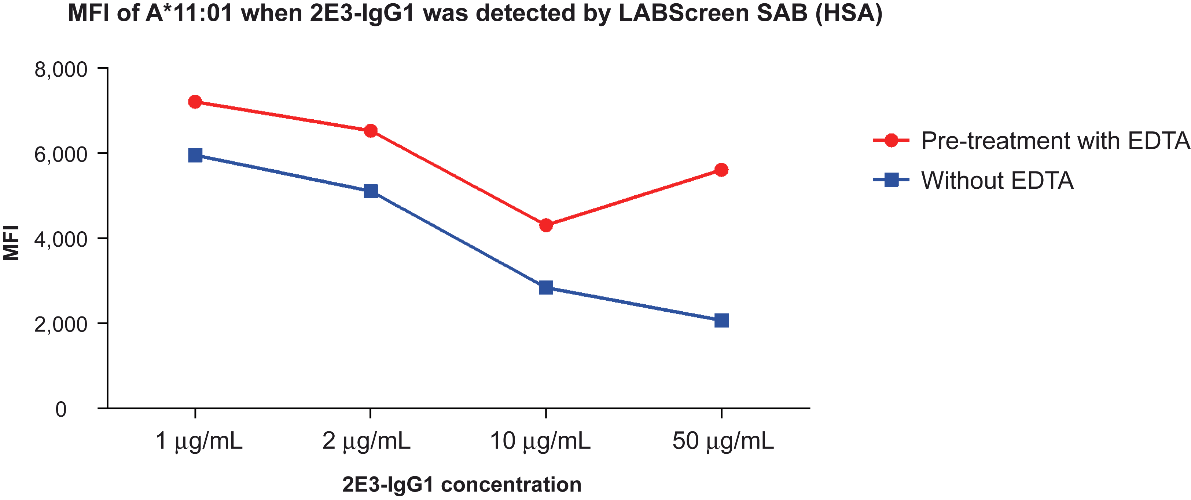
**

**Fig. S3.** Pre-treatment with EDTA did not fully abrogate the prozone effect. Recombinantly expressed 2E3-IgG1was tested at 1 μg/mL, 2 μg/mL, 10 μg/mL and 50 μg/mL using the SAB assay. MFI values of A*11:01 were compared between samples treated with EDTA were and those without EDTA. Figure illustrates assay performance at HSA. MFI: mean fluorescence intensity. HSA: Heath Sciences Authority, Singapore. SAB: single antigen beads. HLA: human leukocyte antigen. EDTA: ethylenediaminetetraacetic acid.
